# Stigmatizing attitudes toward mental disorders among non-mental health nurses in general hospitals of China: a national survey

**DOI:** 10.3389/fpsyt.2023.1180034

**Published:** 2023-08-01

**Authors:** Li Li, Shurong Lu, Chunyan Xie, Yamin Li

**Affiliations:** ^1^Xiangya School of Nursing, Central South University, Changsha, China; ^2^Clinical Nursing Teaching and Research Section, the Second Xiangya Hospital, Central South University, Changsha, China; ^3^Department of Urology, the First Affiliated Hospital of Xinjiang Medical University, Urumqi, China; ^4^Centre for Mental Health | Melbourne School of Population and Global Health The University of Melbourne, Melbourne, VIC, Australia

**Keywords:** stigma, nurse, mental disorder, survey, China

## Abstract

**Background:**

Negative attitudes of nurses toward mental disorders have been reported in various countries. Nurses’ stigmatizing attitudes can harm patients with mental disorders (PWMD), thereby delaying the provision of help to patients and leading to decreased quality of care. In this study, we aimed to assess Chinese nurses’ stigmatizing attitudes toward patients with mental illness and provide a basis for future development and testing of appropriate and culturally adapted interventions to reduce it.

**Objective:**

This study aimed to assess the attitudes of Non-mental Health Nurses (NMHNs) in general hospitals in China toward the stigma of PWMD and determine the factors influencing them.

**Methods:**

A cross-sectional survey of NMHNs in general hospitals were conducted. A self-designed WeChat-based questionnaire was used that included demographic information about the need for training on mental health issues. Participants were provided with a vignette of a depression case with suicidal thoughts. The Depression Stigma Scale (DSS) and Social Distance Scale (SDS) were used to assess attitudes toward mental disorders. Nine questions on the adequacy of knowledge about anxiety and depression and the current status of scale use were used to assess the current status of training needs for mental disorders. Descriptive analysis, chi-square test, and multivariate logistic regression were used for the table.

**Results:**

A total of 8,254 nurses in non-mental health professions participated in this study. The mean DSS score of NMHNs was (17.24 ± 6.700), and the SDS score was (10.34 ± 3.154). The total detection rate of stigma among the survey respondents was 13.40% (1,107/8254). Multivariate logistic regression showed that age between 30 and 39 years [*p* = 0.001, OR = 1.427 (1.154–1.764)], 4 years of work experience and above [*p* = 0.018, OR = 1.377 (1.056–1.796)], having a bachelor’s degree [*p* < 0.001, OR = 0.742 (0.647–0.851)], adequate psychological knowledge [*p* < 0.001, OR = 1.567 (1.364–1.799)], full knowledge of communication with patients with anxiety and depression [*p* < 0.001, OR = 1.848 (1.389–2.459)], and the need to acquire skills to identify anxiety and depression were the influencing factors associated with stigma [*p* < 0.001, OR = 0.343 (0.236–0.499)].

**Conclusion:**

Stigmatizing attitudes toward PWMD exist among NMHNs in general hospitals in China. Thus, more mental health education programs for NMHNs are needed. Factors associated with higher morbidity stigma can be used to develop appropriate interventions to improve NMHNs’ stigmatizing attitudes and provide better quality care to PWMD.

## Introduction

More than 450 million people suffer from mental disorders worldwide (1, 2), but stigmatizing attitudes toward people with mental disorders (PWMD) are prevalent in the global population (3–6). This is also evident in Chinese culture (7–10). Stigma is “the situation of an individual disqualified from full social acceptance” (11). It reflects an individual’s negative attitude/behavior toward mental disorders (12) and is often divided into personal and perceived stigma. Personal stigma is generally the negative attitudes formed by negative perceptions and emotional experiences of PWMD. Meanwhile, perceived stigma is the derogatory behavior toward and rejection of PWMD by others (13). PWMD can be perceived as dangerous, unpredictable, intellectually disable (3), or personally weak (9, 14–16). Stigmatizing attitudes of PWMD may negatively affect their treatment-seeking behavior, education, social activities, work, and mental health (17). Stigma associated with mental disorders is a significant barrier to accessing mental health services and timely treatment (18–20).

Stigma is common among medical caregivers (19, 21, 22). Such stigmatizing attitudes create severe barriers to good patient-provider communication and quality therapeutic care (19, 23, 24), thereby resulting in delayed help-seeking (24), treatment interruptions, safety concerns (19), and poor prognosis (25, 26). Negative attitudes resulting from caregivers’ lack of experience and knowledge about mental disorders can negatively affect patient interactions and the quality of care (19, 27). Stigma can also negatively affect caregivers’ willingness to seek help or disclose mental health problems (19), and nurses’ stigmatizing attitudes may affect other members of the team and future practitioners (28, 29).

The vital link between stigmatizing attitudes, substance use, and suicidal behavior cannot be ignored, such as cannabis being a relevant risk factor linked to suicidal attempts and behaviors (30).

Medical personnel have the closest contact with patients, and they often encounter patients with mental health problems or psychological crises (31). Fewer than 5% of patients see a psychiatrist first, and more than 70% see a non-mental health professional in general hospitals first (32). Due to these factors, non-mental health nurses in general hospitals are highly likely to come into contact with PWMD and play an essential role in the timely referral of patients to psychiatrists (33). However, nurses cannot better provide appropriate assistance to this population because of their negative attitudes toward mental disorders, lack of identification of PWMD, or lack of skills to assist (34, 35), which further affects the effective treatment, early diagnosis, and effective referral rate of this group of patients, thereby resulting in more extended hospital stays, and patients with mental problems being more prone to doctor-patient disputes (36).

Most published studies have focused on the general public’s attitudes toward mental disorders and people with lived experiences (21). However, there is less research on the extent of stigmatization of people with clinically common anxiety and depression by NMHNs in general hospitals. An Australian study comparing the attitudes of health professionals with those of members of the general community showed that health professionals’ stigmatizing attitudes were comparable to those of members of the general community (37). In addition, a study on the attitudes of Finnish nurses showed positive attitudes (38). A recent study of medical students in 65 countries showed that women and medical and nursing students showed more positive attitudes toward PWMD (22). Meanwhile, a survey of healthcare professionals in Qatar showed that nurses had higher levels of stigma than doctors (25). Moreover, studies in Saudi Arabia and Poland showed that stigmatizing attitudes toward PWMD were common among physicians in tertiary care hospitals (39, 40).

Furthermore, a study in Iran showed that stigmatizing attitudes were higher among internal medicine and cardiology departments than among residents in psychiatry (41). Recently, a Greek study showed that healthcare professionals’ willingness to interact with psychiatric patients is relatively poor, and the prejudice against them is high (42).

In addition, screening for depression in general hospitals can improve the accuracy of detecting depressed patients (43) and the overall treatment of patients (44). Nurses are essential in screening, identification, and referral programs for depression in general hospitals’ physical disease management teams (45, 46). A nurse-based model for early screening of depression in patients with physical illness in general hospitals has been proposed in Korea (46), and there are a few reports on such aspects in China.

Therefore, this study primarily aimed to examine the attitudes and frequency distribution of NMHNs toward PWMD and mental disorders among demographic and occupational characteristics in Chinese general hospitals. Secondly, the study aimed to explore factors associated with morbidity stigma and investigate the current use of depression screening by NMHNs, to provide a basis for further intervention studies.

## Methods

### Design and setting

This was a descriptive cross-sectional study to explore the attitudes of NMHNs working in Chinese general hospitals toward PWMD. This study aimed to examine factors associated with the detection rate of stigma and understand the current status of knowledge and skills of anxiety and depression and willingness to use screening scales. A convenience sample was used to collect data. This survey was conducted using a self-designed anonymous WeChat-based questionnaire from April 10 to June 1, 2022. The respondents were NMHNs in general hospitals above the second level in China.

### Ethical clearance

This study was approved by the Ethics Committee of Xiangya Nursing School of Central South University approved on April 20, 2022 (No. E202255). Informed consent was obtained from all participants. This research received grants from the National Natural Science Foundation of China (No. 81873806) and Major Scientific and Technological Projects in Hunan Province (No. 2020SK2085). Completing the survey questionnaire implied consent to join the study.

### Participants’ recruitment and data collection

Firstly, the directors of nursing departments of 14 tertiary-level general hospitals in Xinjiang province were contacted to communicate the considerations related to distributing the questionnaire survey. Then, a link to our questionnaire was sent to the nursing department directors of each hospital via WeChat (social media). Subsequently, the questionnaire link was distributed by the nursing department directors of each hospital to the WeChat groups of the chief nursing officers of each hospital. Afterwards, they separately sent the questionnaire link to the WeChat groups of nurses in their departments. An introduction to the study was displayed on the first page of the questionnaire, and participants selected “agree” to continue the survey or “disagree” to withdraw. Completion of the questionnaire implied consent to join the study. In addition, participants were encouraged to invite colleagues or classmates to participate in the online survey, but no compensation was given. The questionnaire link was distributed among the respondents’ friends and WeChat groups. The sample size was subsequently expanded. Moreover, the study population consisted of front-line nurses working in different departments in Chinese general hospitals above the second level for more than 1 year, excluding nurses specializing in mental health.

The study instruments included the Depression Stigma Scale (DSS) scale, the Social Distance Scale (SDS) scale, and a background questionnaire covering socio-demographic factors, with nine multiple-choice questions on the need for knowledge training and willingness to use the scales for anxiety and depression. Data collection was completed online using WeChat from May 10, 2022, to June 30, 2022. All data were collected anonymously.

### Measures

The survey included sociodemographic information such as gender, age, education level, and occupational questions, including hospital care level, work sector, specialty, title, hours worked, and position. Respondents also answered the following nine questions:

1. What are the most common mental disorders you encounter regularly? (Multiple choice).
2. What are your main ways of obtaining knowledge about mental disorders? (Multiple choice).
3. Do you currently have enough psychological knowledge? (Single-choice question).
4. Do you know how to provide help for people with anxiety or depression? (Single-choice question).
5. Do you think it is necessary to train nurses to recognize anxiety and depression? (Multiple choice).
6. Does your department use anxiety or depression scales for patients? (Single-choice question).
7. Are you willing to use scales to screen patients for anxiety or depression? (Single-choice question).
8. If no, what are the reasons? (Multiple choice).
9. If yes, what are the reasons? (Multiple choice).

### Personal stigma

The present study used the personal depression stigma subscale of the standardized DSS (47, 48). The DSS-Personal Scale consists of nine items scored on a 5-point Likert scale (0 = strongly disagree, 4 = strongly agree) (47). The total score (range 0–36) is calculated by summing all item scores, with higher total scores indicating higher levels of individual morbidity stigma. The DSS-Personal Scale has been widely used in surveys of different populations (3, 10). Our study used the Chinese scale version, which showed excellent psychometric properties (49, 50). The internal consistency of this sample was 0.824. The study analysis combined the categories of agree and strongly agree for each purpose to indicate that the entry had a personal stigma. If there were ≥ 6 entries with personal stigma, we defined that the survey respondent had a personal stigma against mental disorders.

### Social Distance Scale

Willingness to contact the person described in the small case was measured using SDS, a five-item scale developed by Link et al. (51). Each item was rated on a 5-point scale, ranging from absolute willingness (1) to absolute unwillingness (4) (Supplementary material). Our study used the Chinese scale version, which showed good psychometric properties (52). The internal consistency of this sample was 0.886. Lower scores indicate a greater willingness to interact with people with mental illness (for details of the questionnaire in this study, Supplementary material).

### Statistical and data analysis

A total of 8,254 valid questionnaires were collected. In this study, descriptive analyses of participants’ demographic characteristics were performed, reporting numerical variables as means and standard deviations (SD), while categorical data were reported as frequencies and percentages. The “agree” and “strongly agree” options of the DSS-Individual Scale were combined, indicating that the entry had a personal stigma. If ≥6 entries had a personal stigma, we defined the survey participant as having a personal stigma of mental disorders or having no stigma. In addition, the mean and SD of the total score of the DSS-Personal Scale were reported for “most commonly encountered mental disorders,” “main way to obtain knowledge about mental disorders.” “Willingness and unwillingness to use the scale to the four questions of most commonly encountered mental disorders,” “main way to obtain knowledge about mental disorders,” and “willingness and unwillingness to use the scale to screen for causes of anxiety or depression” were reported as numbers and percentages and ranked. Factors associated with the detection rate of stigma were included in subsequent multivariate logistic regression analyses at a prespecified *p*-value of 0.1 to identify significant predictors of the outcome variable having stigma. Associations between the current status of psychological knowledge application and morbidity stigma and social distance were assessed using *t*-tests, *F*-tests, and chi-square tests. All analyses were performed using the IBM software SPSS V.28.0 for Windows. In addition, 95% confidence intervals were used, and all comparisons were two-tailed. The threshold of significance was set at *p* = 0.05.

## Results

### Background characteristics of participants

A total of 8,314 nurses from all provinces in mainland China responded to the questionnaire, of which 60 were excluded because of incomplete data. Of the 8,254 respondents, 95.9% were female (*n* = 7,915), 91.9% worked in a tertiary care hospital (*n* = 7,586), 69.2% held a junior title (*n* = 5,709), 58.7% had a bachelor’s degree (*n* = 4,841), 37.3% had 4–10 years of service (*n* = 3,076), and 8.3% were head nurses (*n* = 687). The respondents’ age ranged from 20 to 59 years, of which 54.0% were between 30 and 39 years old (*n* = 4,469), with a mean age of 33.58 years ±6.823 years. The demographic and training needs of the participants and the distribution of willingness to use the scale are shown in Table 1.

**Table 1:** Demographic characteristics of participants (*n* = 8,254).

Demographic variables		Frequency (n)	Percentage (%)
Level of hospital	Tertiary hospital	7,586	91.9
	Secondary hospital	668	8.1
Work department	Oncology and infection and hemodialysis	1,162	14.1
	Emergency and outpatient	837	10.1
	Intensive care unit	757	9.2
	Internal medicine	2,065	25
	Surgical department	1,653	20
	Gynecology and obstetrics	536	6.5
	Pediatric	303	3.7
	Operating room and anesthesia department and interventional room	406	4.9
	Diagnosis and subsidiary	535	6.5
Gender	Male	339	4.1
	Female	7,915	95.9
Age group (year)	<30	2,434	29.5
	30–39	4,460	54
	≥40	1,360	16.5
Years of occupational experience	1–3	1,052	12.7
	4–10	3,076	37.3
	11–15	2,153	26.1
	≥16	1,973	23.9
Education	Master’s degree or above	135	1.6
	Bachelor’s degree	4,841	58.7
	Associate’s degree or below	3,278	39.7
Professional title	Primary	5,709	69.2
	Intermediate	2,105	25.5
	Senior	440	5.3
Position	Nurse	7,567	91.7
	Head nurse	687	8.3
Do you currently have enough psychological knowledge?	Not enough	4,302	52.1
	Enough	3,952	47.9
Do you know how to provide help for people with anxiety or depression?	Know it completely	659	8
	Know a little	6,574	79.6
	Don’t know	1,021	12.4
Do you think it is necessary to train nurses to recognize anxiety and depression?	Necessary	8,111	98.3
	Not necessary	143	1.7
Does your department use anxiety or depression scales for patients?	Anxiety scale only	700	8.5
	Using only the depression scale	314	3.8
	Using both scales	2,160	26.2
	Not using any scale	5,080	61.5
Are you willing to use scales to screen patients for anxiety or depression	Unwilling	1,362	16.5
	Willing	6,892	83.5

### Association of DSS and SDS scores with nurse characteristics

The mean value of the DSS-Personal Scale for NMHNs was (17.24 ± 6.700), and the SDS score was (10.34 ± 3.154).

The internal consistency and reliability of the DSS and SDS scales were good, with Cronbach alpha values of 0.824 and 0.886, respectively.

Table 2 describes the associations between DSS and SDS mean scores and nurse characteristics. At the bivariate level, DSS mean scores were significantly (*p* < 0.05) related to age, years of experience, level of education, knowledge, knowing ways to communicate with patients with anxiety and depression, having the skills to identify anxiety and depression, and willingness to use scales to screen patients for anxiety and depression. DSS mean scores tend to increase with the increase of age and length of service. Moreover, DSS mean scores tend to decrease with the increase of education levels.

**Table 2:** Scores of DSS and SDS among participants with different characteristics.

Variables		DSS score (mean ± SD)	*T*-/*F*-value	*P*-value	SDS score (mean ± SD)	*T*-/*F*-value	*P*-value
DSS total score (mean ± SD)		17.24 ± 6.700		SDS total score (mean ± SD)		10.34 ± 3.154	
Level of hospital	Tertiary hospital	17.23 ± 6.735	−0.518	0.605	10.37 ± 3.168	2.461	0.014*
	Secondary hospital	17.37 ± 6.284			10.06 ± 2.977		
Work department	Oncology and infection and hemodialysis	16.77 ± 6.543	1.461	0.166	10.12 ± 2.957	1.826	0.067
	Emergency and outpatient	17.41 ± 6.727			10.57 ± 3.137		
	Intensive care unit	17.26 ± 6.879			10.41 ± 3.202		
	Internal medicine	17.24 ± 6.730			10.27 ± 3.169		
	Surgical department	17.21 ± 6.819			10.38 ± 3.278		
	Gynecology and obstetrics	17.63 ± 6.549			10.40 ± 3.063		
	Pediatric	17.94 ± 6.969			10.24 ± 3.172		
	Operating room and anesthesia department and interventional room	17.10 ± 6.285			10.54 ± 3.137		
	Diagnosis and subsidiary	17.31 ± 6.518			10.41 ± 3.156		
Gender	Male	17.16 ± 7.146	−0.217	0.828	10.19 ± 3.404	−0.929	0.353
	Female	17.24 ± 6.680			10.35 ± 3.143		
Age group (year)	<30	16.31 ± 6.709	33.638	0.000**	9.80 ± 3.018	69.970	0.000**
	30–39	17.67 ± 6.765			10.44 ± 3.183		
	≥40	17.46 ± 6.297			11.01 ± 3.139		
Years of experience	1–3	15.67 ± 6.815	26.065	0.000**	9.60 ± 2.933	39.549	0.000**
	4–10	17.19 ± 6.720			10.20 ± 3.199		
	11–15	17.84 ± 6.836			10.47 ± 3.12		
	≥16	17.49 ± 6.318			10.84 ± 3.143		
Education level	Master’s degree or above	15.20 ± 5.404	16.829	0.000**	11.40 ± 2.727	12.323	0.000**
	Bachelor’s degree	16.99 ± 6.450			10.41 ± 3.110		
	Associate’s degree or below	17.68 ± 7.067			10.20 ± 3.224		
Professional title	Primary	17.28 ± 6.894	0.509	0.601	10.13 ± 3.164	43.684	0.000**
	Intermediate	17.17 ± 6.309			10.77 ± 3.062		
	Senior	16.99 ± 5.913			11.07 ± 3.162		
Position	Nurse	17.26 ± 6.773	0.953	0.341	10.29 ± 3.149	−5.571	0.000**
	Head nurse	17.03 ± 5.833			10.99 ± 3.148		
Do you currently have enough psychological knowledge?	Not enough	16.75 ± 6.169	−6.921	0.000**	10.60 ± 3.051	7.545	0.000**
	Enough	17.77 ± 7.196			10.07 ± 3.242		
Do you know how to provide help for people with anxiety or depression?	Know it completely	18.26 ± 8.766	8.338	0.000**	8.85 ± 3.292	121.627	0.000**
	Know a little	17.16 ± 6.557			10.35 ± 3.084		
	Don't know	17.10 ± 5.982			11.27 ± 3.150		
Do you think it is necessary to train nurses to recognize anxiety and depression?	Necessary	17.19 ± 6.662	−3.872	0.000**	10.33 ± 3.139	−2.736	0.000**
	Not necessary	19.86 ± 8.197			11.22 ± 3.857		
Are you willing to use scales to screen patients for anxiety or depression	Unwilling	17.90 ± 6.637	4.012	0.000**	11.39 ± 3.289	13.008	0.000**
	Willing	17.11 ± 6.705			10.14 ± 3.085		

DSS total score (mean ± SD) 17.24 ± 6.700 SDS total score (mean ± SD) 10.34 ± 3.154. *P < 0.05; **P < 0.01.

Analysis for mean scores regarding SDS showed that SDS scores were significantly associated with all variables except department and gender (*p* < 0.05). An increasing trend of SDS scores with age, years of work, education, and title was also found. SDS scores were higher for nurse leaders than for nurses and higher for tertiary hospitals than for secondary hospitals. Not enough psychological knowledge scored higher than enough. Scores ranged from low to high for knowing completely, knowing a little, and not knowing much about providing help to people with anxiety or depression. Those reluctant to train nurses in skills to recognize anxiety and depression scored higher than those willing to train. In terms of using the scale to screen patients for anxiety and depression, those who were reluctant to use it scored higher than those who were willing to use it. Higher scores indicate a greater reluctance to engage and interact with people with mental illness.

### Social distance

#### The prevalence rate of stigma

Table 3 shows that the mental disorder stigma prevalence rate was 13.41% (1,107/8,254) among the 8,254 validated respondents. The differences in stigma prevalence rates were statistically significant (*p* < 0.05) for age, years of work, education level, job title, knowledge adequacy, perception of having skills to identify anxiety and depression, and willingness to use the scale to screen for anxiety and depression. The prevalence rate of stigma was higher among the respondents aged 30–39 than in other age groups, higher in respondents with 11–15 years of work experience than among the other years of service groups, and higher among nurses than among nurse leaders. Interestingly, the prevalence rate of stigma was higher among respondents with sufficient knowledge of psychology than those with insufficient knowledge. In addition, it was higher among those who did not consider it necessary to acquire the skills to identify anxiety and depression than among those who did. Moreover, it was higher among respondents who were not willing to use the scale to screen for anxiety and depression than among the willing group. Furthermore, it was higher in the respondents who fully knew of the need to help people with anxiety and depression than in the other groups.

**Table 3:** Detection of disease stigma in survey respondents with different characteristics (n, %).

Variable		Total (n, %)	No stigma (n, %)	Stigmatized (n,%)	χ^2^	*P*-value
Level of hospital	Tertiary hospital	7,586 (91.90)	6,562 (86.50)	1,024 (13.50)	0.609	0.435
	Secondary hospital	668 (8.10)	585 (87.60)	83 (12.40)		
Work department	Oncology and infection and hemodialysis	1,162 (14.10)	1,022 (88.00)	140 (12.00)	6.649	0.575
	Emergency and outpatient	837 (10.10)	715 (85.40)	122 (14.60)		
	Intensive care unit	757 (9.20)	646 (85.30)	111 (14.70)		
	Internal medicine	2,065 (25.00)	1,791 (86.70)	274 (13.30)		
	Surgical department	1,653 (20.00)	1,425 (86.20)	228 (13.80)		
	Gynecology and obstetrics	536 (6.50)	463 (86.40)	73 (13.60)		
	Pediatric	303 (3.70)	257 (84.80)	46 (15.20)		
	Operating room and anesthesia department and interventional room	406 (4.90)	359 (88.40)	47 (11.60)		
	Diagnosis and subsidiary	535 (6.50)	469 (87.70)	66 (12.30)		
Gender	Male	339 (4.10)	286 (84.40)	53 (15.60)	1.504	0.220
	Female	7,915 (95.90)	6,861 (86.70)	1,054 (13.30)		
Age group (years)	<30	2,434 (29.50)	2,179 (89.50)	255 (10.50)	30.403	0.000**
	30–39	4,460 (54.00)	3,783 (84.80)	677 (15.20)		
	≥40	1,360 (16.50)	1,185 (87.10)	175 (12.90)		
Years of occupational experience	1–3	1,052 (12.70)	954 (90.70)	98 (9.30)	30.249	0.000**
	4–10	3,076 (37.30)	2,676 (87.00)	400 (13.00)		
	11–15	2,153 (26.10)	1,804 (83.80)	349 (16.20)		
	≥16	1,973 (23.90)	1,713 (86.80)	260 (13.20)		
Education	Master’s degree or above	135 (1.60)	125 (92.60)	10 (7.40)	25.292	0.000**
	Bachelor’s degree	4,841 (58.70)	4,256 (87.90)	585 (12.10)		
	Associate’s degree or below	3,278 (39.70)	2,766 (84.40)	512 (15.60)		
Professional title	Primary	5,709 (69.20)	4,912 (86.00)	797 (14.00)	5.830	0.054
	Intermediate	2,105 (25.50)	1,842 (87.50)	263 (12.50)		
	Senior	440 (5.30)	393 (89.30)	47 (10.70)		
Position	Nurse	7,567 (91.70)	6,530 (86.30)	1,037 (13.70)	6.701	0.010*
	Head nurse	687 (8.30)	617 (89.80)	70 (10.20)		
Perceived psychological knowledge	Not enough	4,302 (52.10)	3,849 (89.50)	453 (10.50)	64.250	0.000**
	Enough	3,952 (47.90)	3,298 (83.50)	654 (16.50)		
knowledge and skills in helping people with anxiety or depression	Know it completely	659 (8.00)	512 (77.70)	147 (22.30)	51.969	0.000**
	Know a little	6,574 (79.60)	5,725 (87.10)	849 (12.90)		
	Don't know	1,021 (12.40)	910 (89.10)	111 (10.90)		
Do you think it is necessary to train nurses to recognize anxiety and depression?	Necessary	8,111 (98.30)	7,048 (86.90)	1,063 (13.10)	37.754	0.000**
	Not necessary	143 (1.70)	99 (69.20)	44 (30.80)		
Are you willing to use scales to screen patients for anxiety or depression	Unwilling	1,362 (16.50)	1,157 (84.90)	205 (15.10)	3.777	0.000**
	Willing	6,892 (83.50)	5,990 (86.90)	902 (13.10)		

*P < 0.05; **P < 0.01.

#### Multifactorial logistic regression analysis of the prevalence rate of stigma in DSS

Table 4 shows that the presence or absence of stigma was detected as the dependent variable. The variables with *p*-values greater than 0.2 in Table 3 (nine factors) were included in a multifactorial logistic regression model for analysis, including age, years of experience, education level, professional title, position, adequacy of knowledge, the necessity of skills to identify anxiety and depression, willingness to use the scale to screen for anxiety and depression, and whether or not they were aware of assisting patients with anxiety and depression. Results showed that 30–39 years of age, 4 years or more of work experience, a bachelor’s degree, adequate knowledge, full knowledge of providing help to patients with anxiety and depression, and the need for skills to identify anxiety and depression were the factors influencing the prevalence rate of stigma of the interviewed nurses.

**Table 4:** Predictors of stigmatizing attitudes toward people with mental disorders.

Associated factor		OR (95% CI)	*P*-value
Age group (years)	<30	Reference	
	30–39	1.427 (1.154–1.764)	0.001**
	≥40	1.364 (0.97–1.919)	0.074
Years of occupational experience (years)	1–3	Reference	
	4–10	1.377 (1.056–1.796)	0.018*
	11–15	1.714 (1.247–2.356)	<0.001**
	≥16	1.546 (1.065–2.243)	0.022*
Education	Associate’s degree or below	Reference	
	Bachelor’s degree	0.742 (0.647–0.851)	<0.001**
	Master’s degree or above	0.595 (0.306–1.159)	0.127
Professional title	Primary	Reference	
	Mid-level	0.864 (0.714–1.046)	0.133
	Senior	0.858 (0.574–1.285)	0.458
Position	Head nurse	Reference	
	Nurse	1.168 (0.867–1.572)	0.308
Psychological knowledge	Not enough	Reference	
	Enough	1.567 (1.364–1.799)	<0.001**
Knowledge and skills in help people with anxiety or depression	Don't know	Reference	
	Know a little	1.111 (0.895–1.38)	0.341
	Know it completely	1.848 (1.389–2.459)	<0.001**
Do you think it is necessary to train nurses to recognize anxiety and depression?	Not necessary	Reference	
	Necessary	0.343 (0.236–0.499)	<0.001**
Are you willing to use scales to screen patients for anxiety or depression	Unwilling	Reference	
	Willing	0.935 (0.789–1.108)	0.439

*P < 0.05; **P < 0.01.

#### Current status of training needs and willingness to use scale screening for anxiety and depression

**Figure 1:**
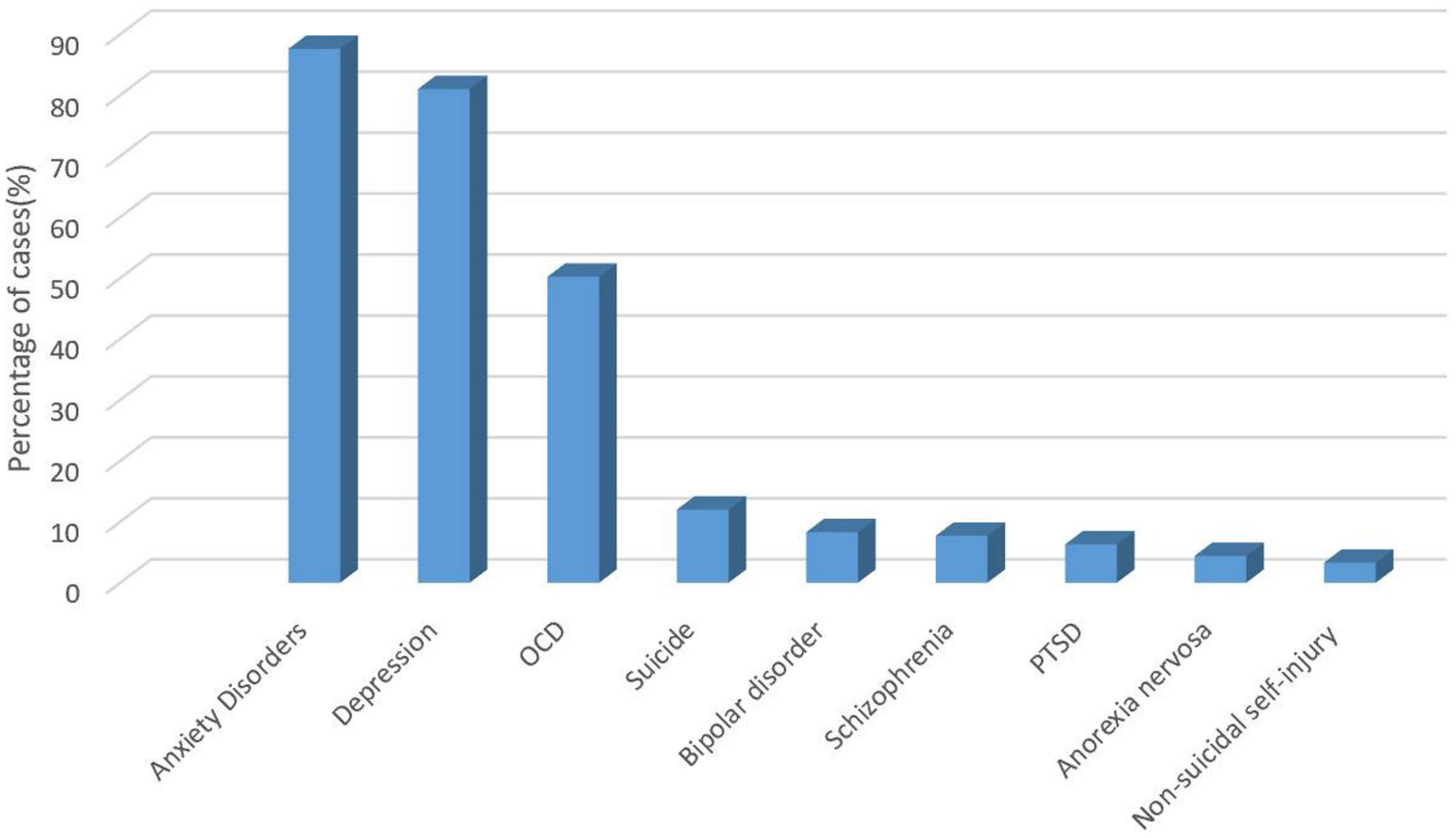
The most common mental disorders encountered regularly.

The most common mental disorder encountered by respondents in this survey was anxiety disorder (87.60%), followed by depression (81.00%) and obsessive–compulsive disorder (50.20%) (Figure 1).

**Figure 2:**
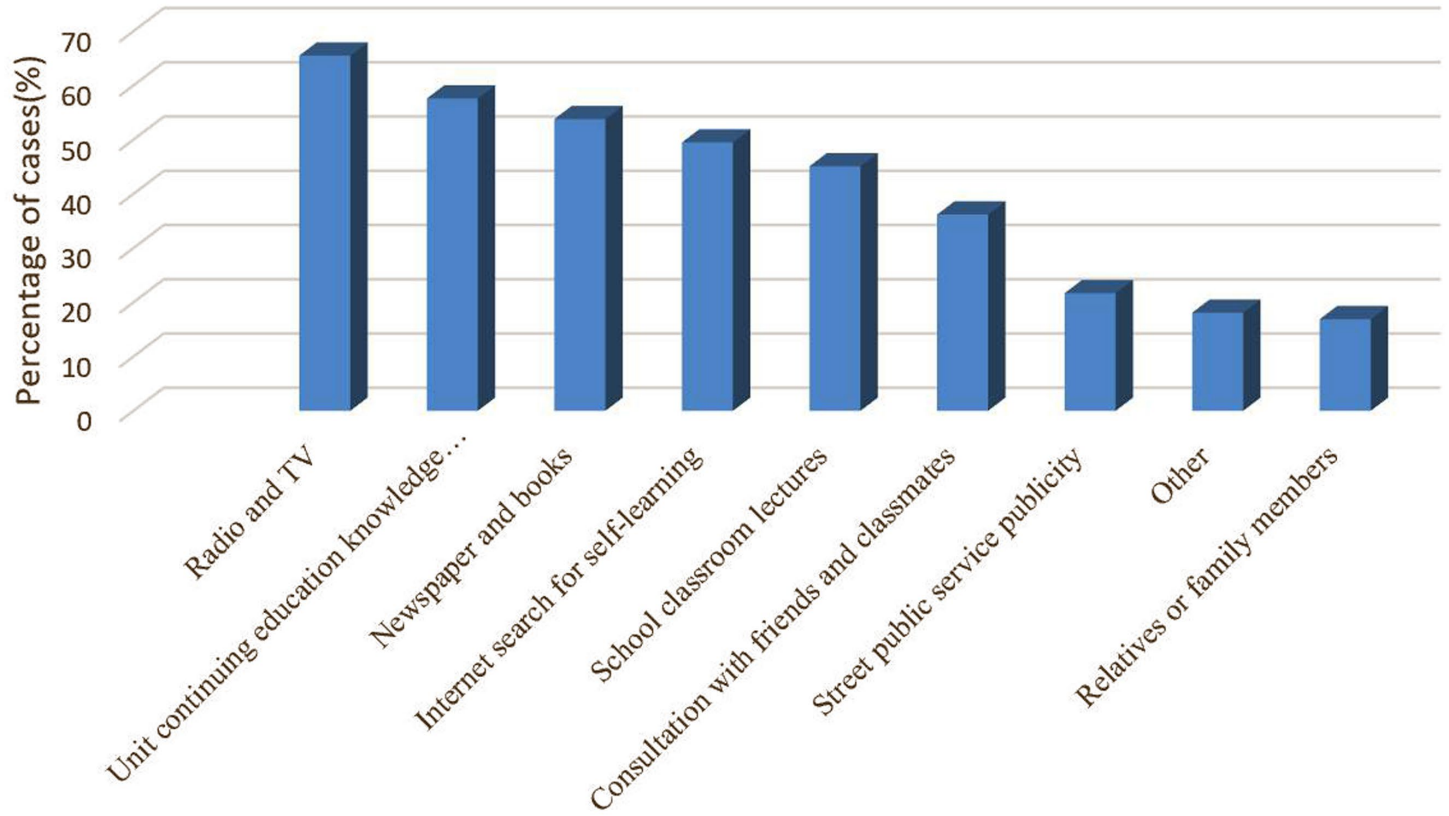
Primary source of knowledge about mental disorder.

The primary source of knowledge about mental disorders for the respondents in this survey was radio and television (87.60%), followed by unit continuing education knowledge lectures and newspapers and books (Figure 2).

**Figure 3:**
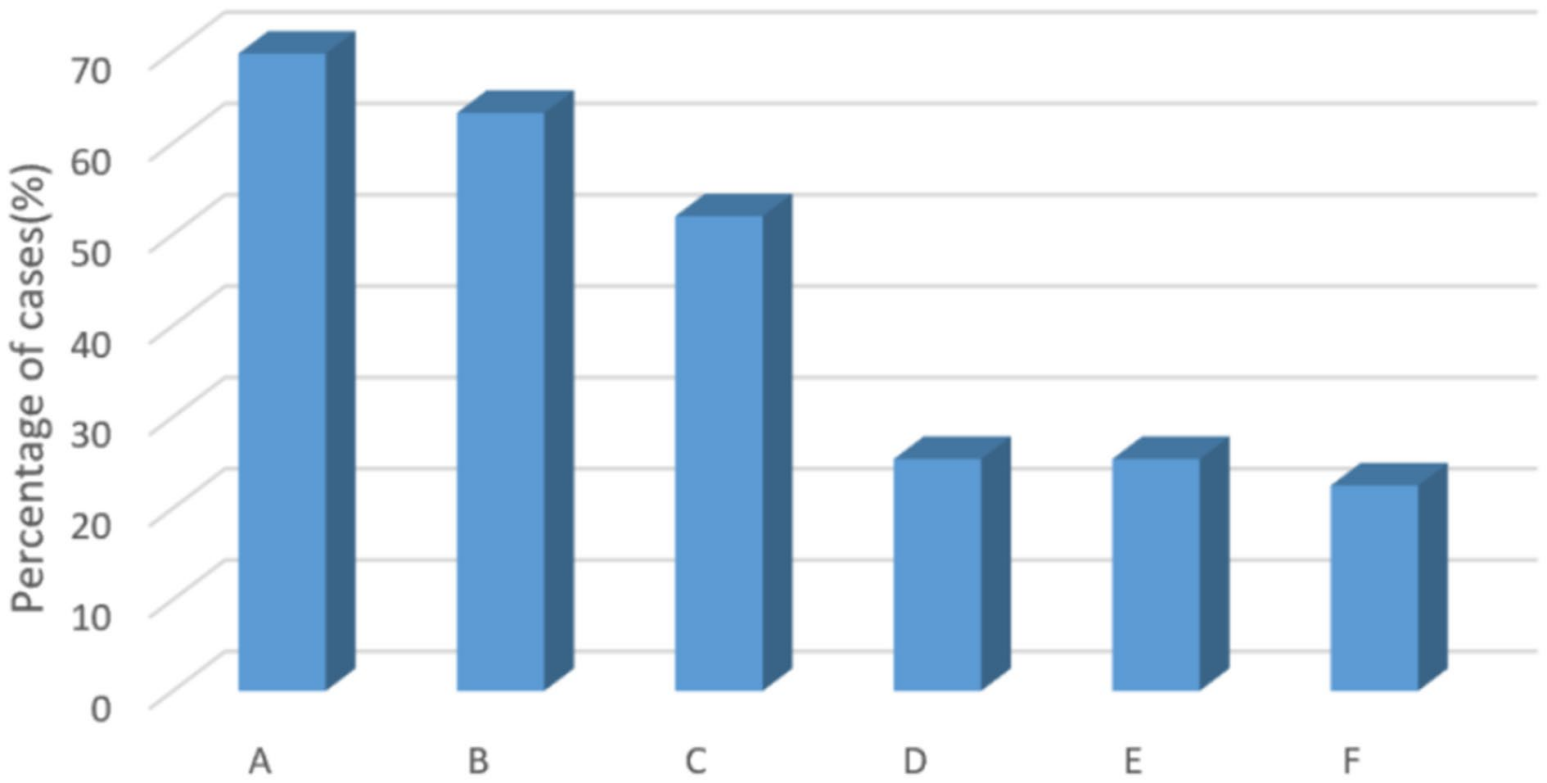
Reasons for reluctance to use scales to screen for anxiety and depression. A, there is no time for screening because of busy work; B, the selected scale takes too long; C, patient refusal; D, this is the doctor’s work area, and the nurse does not need to do this work; E, no need for screening; F, other.

**Figure 4:**
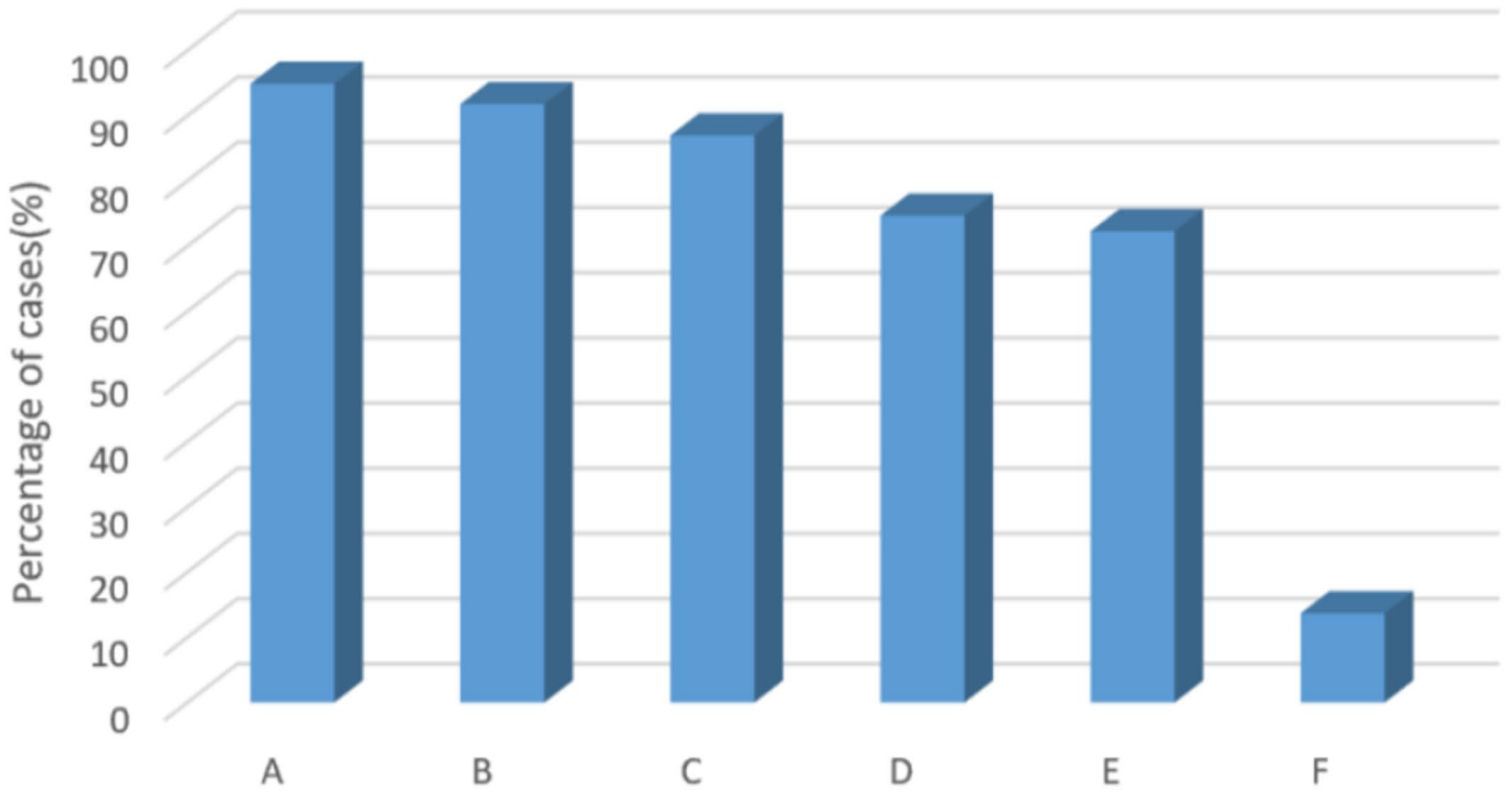
Reasons for willingness to use the scale to screen for anxiety and depression. A, it is conductive to providing better psychosocial care for patients, which can improve treatment compliance and promote early recovery; B, it is beneficial for nurses to find out which patients have anxiety or depression and can focus on them to avoid adverse events; C, knowing more emotional abnormalities of patients, avoiding some doctor-patient disputes, and improving patient satisfaction; D, easy and fast screening using scales; E, it can improve the effective referral rate of patients with psychosomatic abnormalities; F, other.

Figures 3, 4 show that the most popular option for respondents in this survey who were willing to use the scale to screen patients with anxiety and depression was that it would help to provide good psychological care to patients, improve treatment compliance, and promote early recovery (21.8%). Secondly, it helps nurses to know which patients have anxiety symptoms or depressive symptoms and focus on them to avoid adverse events (21.1%). Knowing patients’ psychological abnormalities can improve patient satisfaction and avoid some doctor-patient disputes (20.0%). The number one ranked reason for not wanting to use the scale was being too busy to do a screening. The second ranked reason was the scale chosen needs to be short. The third reason was patient refusal (20.1%). However, the number of people who were willing (83.5%) to use the scale to screen for anxiety and depression was much higher than the number of people who were not (16.5%).

## Discussion

This study found that NMHN in Chinese general hospitals have stigmatized attitudes toward patients with mental disorders. Factors associated with stigma and sources of acquiring knowledge about mental disorders inform the next steps in developing interventions to provide a better quality of care for patients.

This study explored the stigmatization of PWMD in Chinese general hospital NMHNs. In this study, the mean DSS-Personal Scale score of Chinese general hospitals NMHNs was (17.24 ± 6.700) (Table 2), which was higher than the scores of Chinese medical students (13.71 ± 5.350) (10) and Portuguese community adults (12.71 ± 5.520) (53). This finding suggests that the stigma of personal mental disorders is higher in Chinese general hospital NMHNs.

In addition, the survey analysis found that the detection rate of stigma for mental disorders among the nurses interviewed was significantly lower than in studies related to the stigma of patients and families attending outpatient clinics in Chinese domestic community hospitals (21.5%) (54). However, Swedish nurses’ attitudes toward PWMD are similar to those of the general public (55). A study by Cattell showed that nurses had higher negative attitudes toward PWMD than physicians (25), which may indicate a global phenomenon. Media portrayals of mental disorders may influence nurses toward seeing patients as potentially dangerous, unpredictable, violent, or weak (56).

The findings showed an effect of stigma of mental disorders among nurses interviewed between ages 30–39 years [OR = 1.427 (1.154–1.764), *p* = 0.001], which is inconsistent with a study on the stigma of depression in the general population in Canada (57).

In the present study, a bachelor’s degree was also an independent influence on the morbid stigma of mental disorders [OR = 0.742 (0.647–0.851), *p* < 0.001], which is generally consistent with a Greek study (42). Higher levels of education are associated with less stigmatizing attitudes toward PWMD. However, our study showed that having a master’s degree or higher was not an independent influence on the stigma of mental disorders [OR = 0.595 (0.306–1.159), *p* = 0.127]. However, univariate analysis showed that the prevalence rate of stigma gradually decreased with the increase of education level (7.40% for master’s degrees and higher, 12.10% for bachelor’s degrees, and 15.60% for college and lower). Those with a master’s degree had the lowest detection rate of stigma.

Years of work experience is another critical variable in our data. The highest OR for stigma was found among the nurses surveyed with 11–15 years of work experience [OR = 1.714 (1.247–2.356), *p* < 0.001] in a U-shape. Moreover, in the univariate analysis, the stigma prevalence rate was highest among the surveyed nurses with 11–15 years of work experience (16.20%). This may be because most undergraduate medical education programs in China are rarely designed with mental health-related course credit hours (58).

In this study, no independent influences on the stigma of mental disorders were found in the department for NMHNs. However, a study in Qatar showed that stigma was highest among emergency department nurses (25). Individuals requiring acute mental disorder care are initially assessed in the emergency department. Thus, nurses’ attitudes can severely impact the quality of patient care (25). A patient’s physical discomfort may be overlooked and attributed to their mental disorder in the emergency department. This diagnostic masking is a high risk for worsening morbidity and potential mortality. It is a significant barrier to help-seeking behavior and may lead to delays in receiving necessary help (25). A Swedish study showed that staff in inpatient units had more negative attitudes than staff in outpatient departments (21). Greater exposure to mental disorders and higher knowledge of mental disorders predicted lower personal stigma and social distance (59).

This study showed that Chinese NMHNs’ overall mean SDS score was (10.34 ± 3.154). Results also found that the willingness to interact with patients with mental illness was highest among young respondents, those with only a few years of experience, those with low education and title, and who were working in secondary hospitals. Interestingly, we found that the willingness to interact with patients with mental illness was lower for head nurses than for nurses. However, stigma prevalence rates were higher for nurses than for nurse leaders. This finding suggests that a high level of contact with patients with mental illness is not necessarily associated with sufficient willingness to interact with these patients, nor does it decisively reduce existing bias (42, 55).

Therefore, results in Figure 1 indicate that people between the ages of 30–39, with 11–15 years of work experience, with college degrees or less, who believe they have sufficient knowledge and are fully capable of assisting people with anxiety and depression, and who do not believe it is necessary to acquire the skills to identify anxiety and depression, can be the focus of future stigma education and training implementation in the region.

### Current status of training needs and willingness to use scales for anxiety and depression

In this study, respondents commonly encountered anxiety disorders (87.60%) and depression (81.00%). This is consistent with a recent epidemiological study in which anxiety disorders were the most common mental disorder (60). However, most people suffering from anxiety or mood disorders do not immediately seek help from a mental health professional but initially seek help at their local general hospital (61). In contrast, NMHNs are the healthcare workers with the most contact with patients in clinical practice. The ability of these NMHNs to identify and refer patients and their attitude toward them is very important, particularly those patients with non-psychotic disorders who do not receive timely referrals or appropriate treatment. Until they can receive timely referrals, the high expenditure on health resources increases the financial burden on patients and takes away from limited healthcare resources. Stigmatizing attitudes toward these patients can further inhibit their treatment-seeking behavior and affect healthcare quality (62). The poor quality of care they receive can further worsen treatment adherence, reduce the stability of mental disorders, shorten their life expectancy, and increase nurse–patient conflict (63).

Interestingly, although the results of this study showed that the number of people willing to use the scale to screen for mental problems was overwhelming (83.5%, Table 1), the rate of stigma detection among those who were unwilling to use the scale (15.1%) was higher than the rate of stigma detection among those who were willing (13.1%, Table 3), that is, stigma among the unwilling was higher than those willing. This also suggests that respondents unwilling to use the scale to screen patients with anxiety and depression are priority targets for intensive stigma education. Early identification of high-risk patients with mental problems facilitates healthcare professionals to provide more accurate treatment services to such patients. The survey results regarding willingness to use the scale may provide meaningful guidance for developing future management strategies for rapidly identifying patients with mental problems.

In this survey, radio and television were the most common sources of mental health knowledge for the nurses interviewed, followed by continuing education knowledge lectures in the unit and then newspapers and books, slightly different from the study by Wu et al. (33). It is suggested that Chinese nurses have inadequate education and training on the most common mental disorders. Mental health resources, particularly human resources, are inadequate worldwide, especially in low- and middle-income countries (64). NMHNs play an essential role in the identification and timely referral of PWMD. The current survey showed that most surveyed nurses (61.5%) had not used screening tools for anxiety and depression. However, most (83.5%) were willing to use screening tools to identify depression in patients with physical illnesses.

## Strengths and limitations of the study

This study is the first for NMHNs in China, and results reveal a critical public health issue. Therefore, we innovatively used the stigma detection rate method to explore and analyze the factors that influence the stigma of mental disorders in NMHNs, opening up new research ideas in this field. Second, the sample size of this investigation was relatively large for this type of study. However, this study also has its limitations. First, this study was a national online cross-sectional survey. Although 8,254 nurses from all provinces of mainland China responded, the sample was small and disproportionately distributed compared to the number of nursing staff in China, which may affect the validity and generalizability of our findings. Second, no causal relationship can be inferred from the current study, and future longitudinal studies are needed. Third, in the research analysis, the categories of absolute and possible unwillingness for each item in the SDS scale were not combined; this research will be conducted later. The investigation was conducted during the COVID-19 pandemic. Therefore, we could not assess whether or not personal mental health problems and occupational exhaustion affected participants’ responses. Fourth, the current study analyzed self-reported data from NMHN, which may pose a potential risk to the validity of the measurements. However, based on the critical influencing factors of stigma identified in this research, it may provide meaningful guidance for future efforts to improve intensive education on attitudes toward mental disorders appropriate for NMHNs in Chinese general hospitals.

In clinical practice, biased perceptions may negatively affect how patients with mental illness are treated during hospitalization (42, 65). Nurses are the frontline healthcare workers with the most access to patients; thus, they are the most proportional and widespread force in the healthcare workforce and can play an essential role in anti-stigma efforts (66), more so as healthcare workers. Therefore, appropriate educational programs can improve perceptions of mental illness and of patients (67, 68). Our findings suggest that using stigma detection rate methods can detect people at high risk for stigmatizing attitudes and focus on giving targeted interventions based on this. The construction of the mental health system is a significant public health, livelihood, and social issue related to economic and social development and the physical and mental health of the people in China. The reform of China’s mental health system should not only focus on technology and infrastructure but also on improving the way and attitude of providing services to each patient to meet current mental health service needs.

## Conclusion

This study identified a high stigma toward and social distance from mental disorders among NMHNs in Chinese general hospitals and found various associated factors. There were more stigmatizing attitudes among such respondents who were between 30 and 39 years old, had been working for 11–15 years, had a bachelor’s degree, self-identified as having sufficient knowledge of psychology, and thoroughly understood how to communicate with people suffering from anxiety and depression.

The current study also found that anxiety and depression were the most common mental disorders encountered by nurses interviewed, and radio and television were the most common sources of mental health knowledge. The most frequent options for willingness to use the scale to screen for anxiety and depression were facilitating good patient psychological care, improving treatment adherence, and promoting early recovery. In addition, the most frequent reason for being unwilling to use the scale was being too busy at work to do it. The number of people willing to use the scale to screen for anxiety and depression was much higher than those who were not willing to do so. These findings guide the development and implementation of effective interventions for stigma to reduce problems in clinical services in the future.

## Data availability statement

The raw data supporting the conclusions of this article will be made available by the authors without undue reservation.

## Ethics statement

The studies involving human participants were reviewed and approved by The Ethics Committee of Xiangya Nursing School of Central South University approved this study on April 20, 2022 (No. E202255). Informed consent was obtained from all participants. Written informed consent for participation was not required for this study in accordance with the national legislation and the institutional requirements.

## Author contributions

LL and YL participated in the conception and design of this study. CX organized the database. LL performed the statistical analysis and wrote the first draft of the manuscript. SL revised the manuscript. YL and SL provided advice on the statistical analysis and interpretation of the results and reviewed the manuscript draft. All authors contributed to the article and approved the submitted version.

## Funding

This study was supported by the National Natural Science Foundation of China (No. 81873806) and Major Scientific and Technological Projects in Hunan Province (No. 2020SK2085).

## Conflict of interest

The authors declare that the study was conducted without any commercial or financial relationship, which may be considered a potential conflict of interest.

## Publisher’s note

All claims expressed in this article are solely those of the authors and do not necessarily represent those of their affiliated organizations, or those of the publisher, the editors and the reviewers. Any product that may be evaluated in this article, or claim that may be made by its manufacturer, is not guaranteed or endorsed by the publisher.

## Abbreviations

NMHNs: Non-mental health nurses
DSS: Depression Stigma Scale
SDS: Social Distance Scale
OCD: Obsessive–compulsive disorder
PTSD: Post-traumatic stress disorder
PWMD: Patients with mental disorders
GHOC: General hospitals of China
