# Supplementary material for: Stigmatizing attitudes toward mental disorders among non-mental health nurses in general hospitals of China: a national survey
Source: Front Psychiatry. 2023 Aug 1;14:1180034. doi: 10.3389/fpsyt.2023.1180034 (PMC10433212; doi:10.3389/fpsyt.2023.1180034)
Supplement: Supplementary file 1 [file Data_Sheet_1.docx]

**Additional file 1:** Details of the questionnaire in the study.

Thank you for participating in this survey. It take about 3 minutes to finish this anonymize questionnaire, your information will be kept to confidential, you can choose participants chose “agree” to continue or “disagree” to quit.

**Demographic information**

1. Gender: *

| ○male | ○female |  |  |  |  |  |  |
| --- | --- | --- | --- | --- | --- | --- | --- |

2. Age：*

_________________________________

3. The level of the hospital you work in*

| ○tertiary |
| --- |
| ○secondary |

1. Which department do you work in your hospital？ *

_________________________________

5. Your professional title *

| ○senior |
| --- |
| ○intermediate |
| ○primary |
|  |

6. Your educational level *

| ○Associate degree or below |
| --- |
| ○Bachelor’s degree |
| ○Master’s degree or above |
|  |

7. How many years have you been working as a healthcare provider？ Years *

8. Your position *

| ○nurse |
| --- |
| ○Head Nurse |

**Current status of training and scale use for mental disorders.**

1. Which are the most common mental disorders you encounter regularly? (Multiple choice)*.

① Obsessive-compulsive disorder (OCD)

② Suicide

③ Bipolar disorder

④ Anxiety disorder

⑤ Depression

⑥ Anorexia nervosa

⑦ Schizophrenia

⑧ Non-suicidal self-injury (or self-harm)

⑨ Post-traumatic stress disorder (PTSD)

1. What are your main ways of obtaining knowledge about mental disorders? (Multiple choice)*.

① School classroom lectures

② Unit continuing education knowledge lectures

③ Newspaper and books

④ Radio and TV

⑤ Street public service publicity

⑥ Friends and classmates

⑦ Relatives

⑧ Internet search for self-learning

⑨ Other

1. Do you currently have enough psychological knowledge? (Single-choice question)*

① Not enough

② Enough

1. Do you know how to provide help for people with anxiety or depression? (Single-choice question)*

① Know it completely

② Know a little

③ Don't know

1. Do you think it is necessary to train nurses to recognize anxiety and depression? (Multiple choice)*

① Necessary

② Not necessary

1. Does your department use anxiety or depression scales for patients? (Single-choice question)*

① Anxiety scale only

② Using only the depression scale

③ Using both scales

④ Not using any scale

1. Are you willing to use scales to screen patients for anxiety or depression? (Single-choice question)*

① Unwilling

② Willing

1. If no, what are the reasons? (Multiple choice)*

①Patient refusal

②The selected scale takes too long

③There is no time for screening because of busy work

④This is the doctor's work area, and the nurse does not need to do this work

⑤No need for screening

⑥Other

1. If yes, what are the reasons? (Multiple choice)*

① It is conducive to providing better psychosocial care for patients, which can improve treatment compliance and promote early recovery

② The dosage form screening is convenient and quick

③ It is beneficial for nurses to find out which patients have anxiety or depression and can focus on them to avoid adverse events

④ It can improve the effective referral rate of patients with psychosomatic abnormalities

⑤ Knowing more emotional abnormalities of patients, avoiding some doctor-patient disputes, and improving patient satisfaction

⑥ Other

Case 1 describes a fictional person named Zhang Hao, whose current situation is as follows.

Zhang Hao, male, 30 years old, has been depressed for the last few weeks and feels very sad and upset. Despite constantly feeling tired, he has difficulty sleeping almost every night and has lost his appetite and weight. He had trouble concentrating at work, hesitated to make decisions, and was not even able to cope with his daily work, which attracted the attention and concern of his leaders. Zhang Hao felt he would never be happy again and thought his family would be better off without him. He is very desperate and keeps thinking about how to end his life. Please answer the following questions.

| **1**3：The next few questions contain statements about John’s problem. Please indicate how strongly YOU PERSONALLY agree or disagree with each statement. * | | | | | |
| --- | --- | --- | --- | --- | --- |
|  | Strongly agree | Agree | Neither agree nor disagree | Disagree | Strongly disagree |
| a. John could snap out of it if he wanted. |  |  |  |  |  |
| b. John’s problem is a sign of personal weakness. |  |  |  |  |  |
| c. John’s problem is not a real medical illness. |  |  |  |  |  |
| d. John is dangerous. |  |  |  |  |  |
| e. It is best to avoid John so that you don’t develop this problem yourself. |  |  |  |  |  |
| f. John’s problem makes him unpredictable. |  |  |  |  |  |
| g. You would not tell anyone if you had a problem like John’s. |  |  |  |  |  |
| h. I would not employ someone if I knew they had a problem like John’s. |  |  |  |  |  |
| i. I would not vote for a politician if I knew they had suffered a problem like John’s. |  |  |  |  |  |

| **14**  The following questions ask how you would feel about spending time with John. Would you be happy * | | | | |
| --- | --- | --- | --- | --- |
|  | Yes, definitely | Yes, probably | Probably not, or | Definitely not |
| a. To move next door to John? |  |  |  |  |
| b. To spend an evening socializing with John? |  |  |  |  |
| c. To make friends with John? |  |  |  |  |
| d. To work closely with John on a project at work? |  |  |  |  |
| e. To have John marry into your family? |  |  |  |  |
